# Natural and Experimental Persistence of Highly Pathogenic H5 Influenza Viruses in Slurry of Domestic Ducks, with or without Lime Treatment

**DOI:** 10.1128/AEM.02288-20

**Published:** 2020-11-24

**Authors:** Audrey Schmitz, Marion Pertusa, Sophie Le Bouquin, Nathalie Rousset, Katell Ogor, Marie-Odile LeBras, Claire Martenot, Patrick Daniel, Ana Belen Cepeda Hontecillas, Axelle Scoizec, Hervé Morin, Pascale Massin, Béatrice Grasland, Eric Niqueux, Nicolas Eterradossi

**Affiliations:** a Ploufragan-Plouzané-Niort Laboratory, Avian and Rabbit Virology, Immunology and Parasitology Unit, French National Reference Laboratory for Avian Influenza and Newcastle Disease, French Agency for Food, Environmental and Occupational Health & Safety, Ploufragan, France; b Institut Technique de l’Aviculture, UMT Sanivol, Paris, France; c Ploufragan-Plouzané-Niort Laboratory, Epidemiology, Health and Welfare in Animal Production Unit, French Agency for Food, Environmental and Occupational Health & Safety, Ploufragan, France; d Laboratoire des Pyrénées et des Landes, Mont de Marsan, France; e LABOCEA Site de Ploufragan, Zoopôle, Ploufragan, France; f Grimaud Frères, Sèvremoine, France; Centers for Disease Control and Prevention

**Keywords:** avian influenza, H5HP, duck, slurry, persistence, lime treatment

## Abstract

Infections by A/H5 and A/H7 avian influenza viruses (AIVs) can cause acute disease and are therefore notifiable in poultry and wild birds. During winter 2015-2016, several cases of infection caused by highly pathogenic (HP) AIVs belonging to the A/H5N1, A/H5N2, and A/H5N9 subtypes were detected in southwestern France. Throughout winter 2016-2017, several cases of infections caused mainly by A/H5N8 HP AIV (A/goose/GD/1/1996, clade 2.3.4.4) were detected across Europe. On both occasions, the viruses were widely detected on palmiped farms in France. This study was designed to evaluate the persistence of A/H5 HP AIV in slurry from various duck productions. This was achieved (i) in the laboratory setting by artificially spiking four AIV-free slurry samples with known amounts of A/H5N9 HP AIV and monitoring virus infectivity, with or without lime treatment to achieve pH 10 or pH 12, and (ii) by sampling slurry tanks on five naturally A/H5N8 HP-contaminated farms. Experimental results in artificially spiked slurry suggested virus survival for 4 weeks in slurry from Muscovy or Pekin duck breeders and for 2 weeks in slurry from ducks for foie gras production during the assisted-feeding period, without lime treatment. Persistence of infectious A/H5N9 HP AIV in all slurry samples after lime treatment at pH 10 or pH 12 was less than 1 week. The A/H5N8 HP AIV persisted in naturally contaminated untreated slurry for 7 weeks. The results obtained provide experimental support for the 60-day storage period without treatment or the 7-day interval after lime treatment defined in French regulations for slurry sanitization.

**IMPORTANCE** From November 2015 to July 2017, two successive episodes of H5 highly pathogenic avian influenza viruses (HP AIVs) infections occurred on poultry farms in France, mostly in domestic ducks raised for foie gras production in southwestern France. During the two epizootics, epidemiological investigations were carried out on infected farms and control and biosafety measures were implemented in association with surveillance in order to stop the spread of the viruses. Effluents are known to be an important factor in environmental dissemination of viruses, and suitable effluent management is needed to help prevent the spread of epizootics to other farms or pathogen persistence at the farm level. The present study was therefore designed to assess how long infectious A/H5 HP AIVs can persist in naturally or experimentally contaminated fecal slurry samples from ducks, with or without sanitization by lime treatment.

## Abstract (precis)

From November 2015 to July 2017, two successive episodes of H5 highly pathogenic avian influenza viruses (HP AIVs) infections occurred on poultry farms in France, mostly in domestic ducks raised for foie gras production in southwestern France. During the two epizootics, epidemiological investigations were carried out on infected farms and control and biosafety measures were implemented in association with surveillance in order to stop the spread of the viruses. Effluents are known to be an important factor in environmental dissemination of viruses, and suitable effluent management is needed to help prevent the spread of epizootics to other farms or pathogen persistence at the farm level. The present study was therefore designed to assess how long infectious A/H5 HP AIVs can persist in naturally or experimentally contaminated fecal slurry samples from ducks, with or without sanitization by lime treatment.

## INTRODUCTION

Avian influenza virus (AIV) infections are widespread in many different species of wild and domestic birds. Wild waterfowl (especially in the orders *Anseriformes* and *Charadriiformes*) are the natural reservoir of all currently described AIV subtypes, including 16 different hemagglutinin (H1 to H16) and 9 neuraminidase (N1 to N9) subtypes. These birds usually shed the virus through respiratory aerosols or contaminated feces, without developing symptoms (1, 2). These dissemination routes allow transmission to susceptible naive hosts and contamination of natural environments, especially through contaminated feces. Complex natural environments, such as lake sediment, mud, and sand, have the ability to readily adsorb virus particles (3) and may act as a source of influenza viruses in the aquatic habitat (4–6).

Infection of terrestrial poultry, occurring by direct spillover from wild birds or indirect transmission of AIV, is usually asymptomatic or results in mild respiratory disease or a drop in egg production (7). However, more severe symptoms with high mortality have also been associated with several different epizootics of highly pathogenic (HP) A/H5 and A/H7 AIVs. Therefore, A/H5 and A/H7 AIVs are notifiable to the World Organisation for Animal Health (OIE; http://www.oie.int/animal-health-in-the-world/update-on-avian-influenza/2018/).

From November 2015 to July 2017, two successive episodes of A/H5 HP AIV infections occurred on poultry farms in France, mostly in domestic ducks raised for foie gras production in southwestern France. During winter 2015-2016, 81 outbreaks were reported on poultry farms (mostly domestic waterfowl without overt disease) due to one of the three detected subtypes: A/H5N1, A/H5N2, and A/H5N9 HP AIVs. No infection was detected in wild birds. These A/H5 HP AIVs belonged to an H5 genetic lineage significantly different from the A/goose/Guangdong/1/1996 lineage and therefore do not have an assigned clade number (clades have been defined within the A/goose/Guangdong/1/1996 lineage only [8, 9]). They presented an unusual HP hemagglutinin cleavage site and a high capacity to spread and induced limited clinical signs in ducks (8, 10). During winter 2016-2017, outbreaks due to the A/H5N8 HP AIV from the A/goose/Guangdong/1/1996 lineage (clade 2.3.4.4) among wild birds and poultry were recorded in France and in many countries in Europe (11–14). This A/H5N8 HP AIV induced more obvious clinical signs (nervous symptoms) and higher mortality than the French A/H5 HP AIV detected in 2015-2016. In France, outbreaks affected both poultry (486 cases) and wild birds (55 cases). Domestic ducks raised for foie gras production were again the most affected.

Mule duck farming linked with foie gras production is widespread in southwestern France, which represents the main production area in the country (15). Briefly, this production system entails crossing Pekin female breeder ducks (Anas platyrhynchos) with male Muscovy ducks (Cairina moschata). After 3 to 4 weeks of rearing in a closed barn and controlled environment, the hybrid ducklings are transferred to the open range for a 7- to 11-week rearing period and are then moved to closed barns for a 10- to 14-day assisted-feeding period, before finally being transferred to the slaughterhouse. In most cases, the different rearing steps are performed by specialized farmers, and thus they entail moving the birds from one farm to the next, with specific operational teams and transportation systems involving trucks and crates. The resulting production system therefore combines several aspects: rearing of *Anseriformes* ducks, possible contact with wildlife in the open range, multiple operators and materials, several transfers of animals along the production cycle, and the need to recycle or spread possibly contaminated slurry. It is essential to manage these factors in order to implement efficient biosafety.

During the two epizootics, epidemiological investigations were carried out on infected farms and control and biosafety measures were implemented in association with surveillance in order to stop the spread of the viruses. Effluents can be an important factor in environmental dissemination of viruses. On most breeding and assisted-feeding farms, feces are collected in the poultry house as slurry (liquid mixture of feces and urine added to litter, feed residues, washing water, and rainwater). These slurry effluents are different from solid manure, present in most gallinaceous productions, which is a mixture of feces together with substantial quantities of bedding materials, dense enough to be handled as a solid.

Slurry is stored until processing for energy production (methanization) or spreading on pastures as a fertilizer for agronomic use (approved only at certain times of the year). Proper management of slurry is needed to help prevent the spread of epizootics to farms in the vicinity during slurry spreading on fields for soil enrichment or pathogen persistence at the farm level (16). Various parameters, such as temperature, pH, salinity, humidity, UV light, and physical or chemical composition, can affect virus survival in different liquid environments, such as saline solution, chicken manure, duck feces, duck meat, and lake sediment (4–6, 17–46). A combination of low temperature, neutral pH conditions, and absence of exposure to UV light in a medium enriched with organic material could significantly favor the persistence of infectious AIV particles (4, 6, 17–42, 45, 46). Such conditions prevail in fecal slurry tanks in winter, and it is therefore important to define control measures that could be used to sanitize slurry. Slurry sanitization is usually performed by chemical treatment to modify the pH, using lime, sodium hydroxide, formalin, or peracetic acid (26, 47). In France, only three methods of slurry treatment are officially accepted in the case of HP AIV detection: treatment in a methanization plant, lime treatment, or storage for at least 60 days before slurry spreading (34). However, only limited information was available in the published scientific literature regarding AIV survival in poultry feces (4, 22, 26, 27, 33, 36, 38, 46), and none pertained to ducks or duck slurry.

The present study was therefore designed to assess how long infectious A/H5 HP AIVs can persist in naturally or experimentally contaminated fecal slurry samples from ducks, with or without sanitization by lime treatment. These data are important with regard to measuring the actual efficiency of officially recommended measures for slurry sanitization and helping to prevent further dissemination of the virus through slurry management during epizootics.

## RESULTS

### Experimentally contaminated slurry.

**(i) Physicochemical characteristics.** The physicochemical characteristics of the AIV-free slurries used are presented in Table S1. The four slurries exhibited neutral pH, between 6.1 and 7.3.

**(ii) Experimental spiking of negative slurries with A/H5N9 HP AIV.** Prior to being spiked with A/H5N9 HP AIV, the four slurries used for the experimental contamination study were confirmed as being negative for AIV, as no influenza virus or contaminating hemagglutinating viruses were detected in embryonated eggs and no influenza virus genome was detected by M gene real-time reverse transcription-PCR (M-rRT-PCR). These slurries were therefore considered suitable for spiking with A/H5N9 HP AIV. A positive-control buffer (phosphate-buffered saline [PBS]) spiked with A/H5N9 HP AIV using the same protocol and then stored under similar conditions allowed reisolation of the A/H5N9 HP AI each week until the end of the study (7 weeks postspiking). Sequences of A/H5N9 HP AIV are available in the GISAID database (accession numbers are given in Table S2).

**FIG 1:**
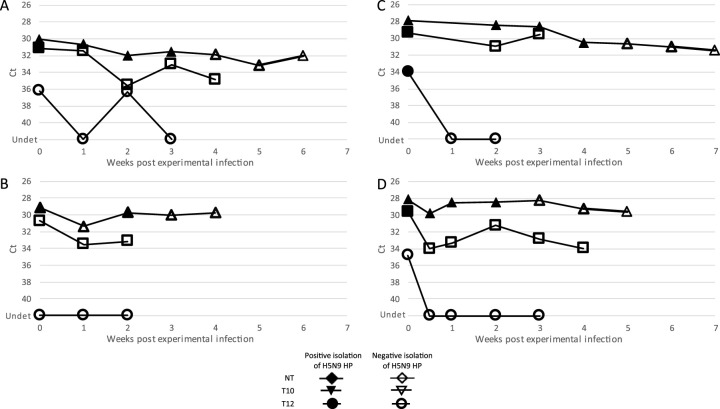
Molecular and virological detection of highly pathogenic H5N9 in four experimentally spiked slurries (A to D), from different production facilities, with or without lime treatment. Triangles, squares, and circles indicate results of M-rRT-PCR from slurry samples without treatment, with lime treatment to achieve pH 10, or with lime treatment to achieve pH 12 (NT, T10, and T12, respectively). Filled and open symbols indicate positive and negative viral isolation of HP H5N9, respectively. The first day of the kinetic study (D0) was the day of artificial contamination and treatment. The standard deviation of the M gene rRT-PCR was estimated as a *C_T_* value of 1.2 (standard *C_T_* values between 31 and 33) using a standard RNA in a series of 20 repeated assays. Ct, cycle threshold; Undet, undetermined.

**(iii) Persistence of infectious A/H5N9 HP AIV in experimentally contaminated slurry.** Figure 1 presents, for each spiked slurry, the results of weekly virus isolation assays and M-rRT-PCRs. All four nontreated spiked slurries allowed A/H5N9 HP AIV isolation between 2 and 4 weeks after spiking, although the M gene could be stably detected for the whole duration of the study, up to 7 weeks after spiking. In the four spiked slurries treated with milk of lime to achieve pH 10, infectious A/H5N9 HP AIV was isolated on the day of contamination only from slurries A, C and D (no isolation for slurry B). Discrepantly, detection of the M gene was stable for the duration of the study for the four spiked pH 10 slurries tested, and the AIV genome amounts detected were in the same range (difference in cycle threshold [*C_T_*] of 2) as those detected in untreated slurries. Following lime treatment to achieve pH 12, only slurry C allowed the isolation of A/H5N9 HP AIV, on the day of spiking only, whereas slurries A, B, and D did not allow virus reisolation. The pH 12 condition induced a strong decrease in M gene detection, as the M gene was mostly detected on the day of spiking only and was undetectable (*C_T_* values > 40) afterwards.

### Naturally contaminated slurry.

**FIG 2:**
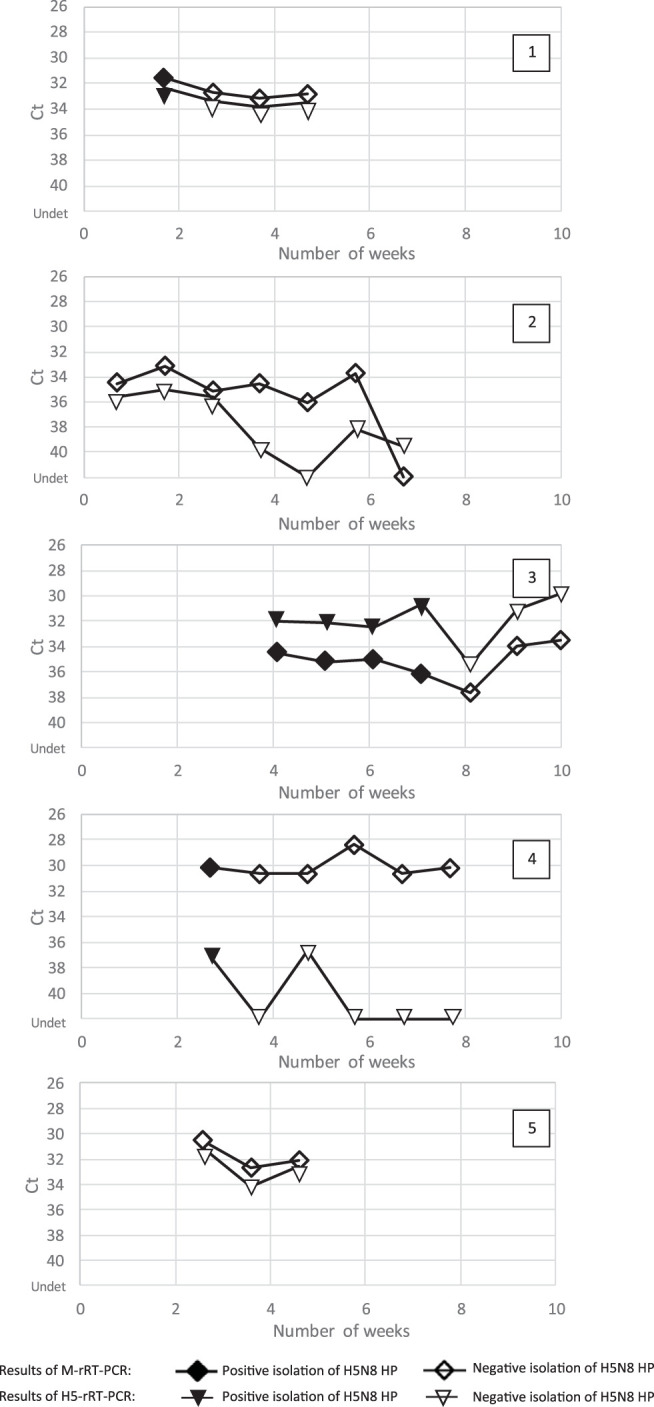
Molecular and virological detection of highly pathogenic H5N8 in naturally contaminated slurries. Diamonds and triangles indicate results of weekly subsampled slurries obtained with M- and H5-rRT-PCRs, respectively. Filled and open signs indicate positive and negative viral isolation of H5N8 HP, respectively. The first day of the kinetic study (D0) was estimated as the last day when fresh fecal material from infected ducks could have been delivered into the slurry tank at the farm level. The standard deviation of the M gene rRT-PCR was estimated to be a *C_T_* value of 1.2 (standard *C_T_* values between 31 and 33) and that for the H5 gene rRT-PCR was a *C_T_* value of 1.3 (standard *C_T_* values between 34 and 36) using a standard RNA in a series of 20 repeated assays. Ct, cycle threshold; Undet, undetermined.

Figure 2 and Table 1 present, for each slurry, the results of weekly virus isolation assays or M- and H5-rRT-PCRs. Slurries 2 and 5 did not result in A/H5N8 HP AIV isolation, although the M or H5 gene could be stably detected for at least 3 weeks. A decrease in genome levels was then observed from 3.7 weeks onwards in slurry 2 (H5 detection). Infectious A/H5N8 HP AIV was isolated from slurries 1, 3, and 4 for 1.7 to 7 weeks. Genome detection by M- and H5-rRT-PCR was stable for the duration of the study for slurries 1 and 3 (maximum of 10 weeks for slurry 3) and for slurry 4 by M-rRT-PCR (with no H5 gene detection in slurry 4 after 5.7 weeks). Infectious viruses other than A/H5N8 HP AIV were also isolated in embryonated eggs. These included an H4 AIV in slurry 2 until 4.7 weeks, avian avulavirus 1 (AAvV-1) in slurries 3 and 5 until the end of the study for these samples (10 weeks and 4.6 weeks, respectively), and AAvV-6 from slurry 4 until the end of the study (7.7 weeks). The two AAvV-1 isolates in slurries 3 and 5 were both avirulent and were characterized as class II, genotype 1. Isolation in embryonated eggs was reiterated with slurries 2 and 5, after neutralization of the H4 or AAvV-1 contaminants, respectively. These assays did not allow reisolation of A/H5N8 HP AIV.

**TABLE 1:** Identification and persistence of isolated viruses other than A/H5N8 HP AIV from naturally infected slurries (no lime treatment) in egg culture

Slurry	Virus isolation
Slurry 1	No isolation of other viruses
Slurry 2	Isolation of H4 AIV until 4.7 wk
Slurry 3	Isolation of AAvV-1 until end of study (10 wk)
Slurry 4	Isolation of AAvV-6 until end of study (7.7 wk)
Slurry 5	Isolation of AAvV-1 until end of study (4.6 wk)

For each slurry, sequences encompassing the cleavage site in the H5 gene of each A/H5N8 HP AIV corresponding to the initial isolates were obtained. Regarding the 201 common nucleotides, sequencing data from slurries 1 to 5 were found to be identical, with only a nucleotide change that did not change the corresponding amino acid. All sequences are available in the GISAID or GenBank database (accession numbers are presented in Table S2).

## DISCUSSION

This study was initiated during the 2015-2016 French H5 AIV epizootic, when the A/H5N9 virus was most prevalent (10). As this virus caused only subclinical infection in ducks (8, 10), the initial contamination at the farm level could not be easily dated, and the study was therefore designed based on the experimental contamination of duck slurry that was kept under laboratory conditions at a temperature mimicking the average winter temperature in southwestern France. The A/H5N9 and other related A/H5N1 and A/H5N2 AIVs (10) were successfully eradicated through massive farm depopulation, mandatory fallowing, and increased sanitization during spring and summer 2016. The 2016-2017 epizootic, due to the HP A/H5N8 AIV, provided the opportunity to complement the experimental A/H5N9 study with the longitudinal follow-up of contaminated farms, as the A/H5N8 virus caused clear clinical signs, allowing us to date the initial introduction of the pathogen at the farm level. The number of naturally infected farms monitored during the course of the second epizootic (*n* = 5) was necessarily limited, due to practical laboratory constraints and the labor intensity of the study protocol, and this precludes any statistical evaluation of the duration of virus survival. Furthermore, for biosafety reasons (depopulation, decontamination, and disinfection processes), only one initial sample was collected on each farm instead of weekly samples (repeated sampling in naturally contaminated slurry tanks would furthermore raise the question of how representative and repeatable the sampling process is at the farm level).

The persistence of infectious AIV in the environment is influenced by various physical and chemical parameters, such as temperature, exposure to UV, pH, presence of chemical agents or detergents, salinity, the nature of the matrix, and the presence of organic material (4–6, 17, 19–25, 27–46). Survival of AIV has also been shown to vary depending on the virus strain (19, 22, 27, 30, 33, 35, 37, 46).

In complex environments with a high content of biological material, such as manure or chicken and duck feces, influenza viruses appear to retain infectivity for shorter periods than in water (4, 17, 19–22, 24, 27, 28, 32, 35–40, 42, 45, 46, 48). Previous published studies specific to persistence of AIV in manure or poultry feces are presented in Table S3.

In the present study, the persistence of A/H5 HP AIV in naturally contaminated or artificially spiked duck slurries was evaluated at 5°C, with and without lime treatment. The kinetic study was carried out on a small volume of slurry (600 ml) which was either naturally infected and collected from an A/H5HP outbreak or experimentally spiked in the laboratory and stored at 5°C under biosafety level 3 (BSL3) controlled conditions. Analyses were performed on weekly subsamples of the stored slurries. This temperature of 5°C was selected as it is representative of the mean winter temperature in southwestern France (http://www.meteofrance.com/climat/france/nouvelle-aquitaine/regin09/normales), which is the largest duck-producing region in the country and which was severely affected by two A/H5 HP AIV epizootics in the winters of 2015-2016 and 2016-2017 (8, 10–14). Additionally, this temperature has previously been described as suitable for virus survival (4–6, 18–21, 23, 24, 28, 32–46, 49), and therefore, this approach allowed us to test a worst-case scenario and obtain a safety margin, as survival in actual conditions may be shorter than that observed in the experimental study. Control of the two A/H5 HP epizootics in France entailed storage of contaminated duck slurry on infected farms, and it was therefore important to evaluate experimentally how long contaminated slurry would remain infectious so as to define the interval required for slurry sanitization. The two pH levels tested in our experimental study, pH 12 and pH 10, correspond to the limits of the pH range targeted when lime treatment was implemented in the field (47, 50) as part of the sanitization program designed for infected farms. As the genome of noninfectious AIV was previously shown to persist in certain environments (48), two complementary methods of virus detection (viral isolation on eggs and rRT-PCR) were implemented in the present study. The A/H5N9 HP AIV used for experimental spiking of slurries was selected for its high capacity to spread, coupled with limited clinical signs observed in ducks. It was proven that this virus had spread silently for several months before it was initially detected (10), and spreading of virus-contaminated duck slurry was hypothesized to be one factor possibly contributing to virus dissemination (16). During the course of the A/H5N9 experimental study, the occurrence of the A/H5N8 HP AI epizootic provided the opportunity to supplement our experimental study with a survey of duck slurries naturally contaminated by another H5 HP AIV strain and similarly stored at 5°C.

Our results with naturally contaminated slurries revealed maximum persistence of infectious A/H5N8 HP AIV at 5°C for 7 weeks, while the monitoring of experimentally spiked slurries stored at 5°C demonstrated maximum persistence of infectious A/H5N9 HP AIV for 4 weeks. The A/H5N9 HP AIV stored similarly but diluted in PBS buffer still proved to be infectious at the end of the experiment (>8 weeks). Consistently with previous findings obtained with ducks and chickens (22, 28, 36, 48), the microbial/bacterial flora and the physicochemical composition of duck slurry thus seemed to significantly reduce the persistence of infectious A/H5N9 HP AIV. There are three main hypotheses to explain the difference in persistence observed in the present study between the natural and experimental contaminations (7 and 4 weeks of survival, respectively). First, the difference in persistence could be due to the composition of slurries collected at different times (winter for naturally contaminated slurries versus spring for experimentally spiked slurries), which could affect in particular the microbiome and physicochemical properties. To evaluate this hypothesis, new samples collected during the same period should be tested. Second, the difference in persistence observed between natural and experimental contaminations could also be due to the A/H5 HP AIV strains studied. It is of course possible that the A/H5N8 HP AIV strain belonging to the A/goose/Guangdong/1/1996 lineage could be more resistant than the A/H5N9 HP AIV European strain. Labadie et al. described essential HA amino acids for AIV persistence in the environment (51). Here, both the A/H5N8 HP and A/H5N9 HP viruses exhibited only the lack of insertion of a K residue at position 147 and the presence of a Y residue at position 543, which are able to induce an increase in the persistence of these viruses in the environment (51). The other amino acid positions listed in that report (residues S at position 53, Q at position 299, and A at position 326 [51]) are not present in the viruses included in the present study. The last hypothesis is that low residual doses of the A/H5N8 HP AIV could more readily infect embryonated eggs than similar amounts of the A/H5N9 HP AIV, leading to an apparently longer survival time of the former virus. A similar experimental study performed with the A/H5N8 HP AIV would be necessary to support either hypothesis formally.

In contrast to virus isolation, which was possible for only 4 (A/H5N9) to 7 (A/H5N8) weeks, the AIV genomes of both viruses could still be detected for 2 to 5 weeks after extinction of infectious virus (slurries B and 4, respectively). The *C_T_* values obtained in rRT-PCR for the M and H5 genes under natural or experimental conditions demonstrate stable persistence of the genome in untreated slurries throughout this period. As a result, whereas AIV genome detection demonstrates the previous presence of AIV, it cannot be used to assume the actual presence of infectious AIV, which must be confirmed with another method such as virus isolation.

Our experimental study also investigated two protocols to sanitize duck slurry: lime treatment at pH 10 and at pH 12. Both treatments strikingly reduced virus persistence. Live virus could be isolated only on the day of spiking at pH 10 and at pH 12 in 3 of 4 and 1 of 4 assays, respectively. In addition, increasing pH directly increased the degradation of the virus genome in the treated slurry: at pH 10, the genome could be stably detected, whereas at pH 12, the genome could no longer be detected as early as 1 week after lime treatment. These results extend previous knowledge on the effects of basic pH on AIV survival in allantoic fluids, as 15 min of treatment at pH 10 or pH 12 was previously shown to have no detrimental effect on AIV hemagglutinating activity (28). Similarly, in another study, an alkaline treatment at pH 11 or pH 13 was virucidal after a 6-h contact time (32).

Interestingly, naturally contaminated slurries presented coinfections with hemagglutinating viruses other than A/H5N8 HP AIV (AAvV-1, AAvV-6, or H4 AIV) that were adventitiously detected upon inoculation of our slurry samples on embryonated eggs. Coinfections are not uncommon and have been found previously on duck farms, making it unsurprising to detect other AIVs or avulavirus (52). Reassortment was demonstrated to be an important evolutive mechanism during the 2015-2016 epizootics (10), and extensive reassortment could have occurred only when duck farms were coinfected by AIVs belonging to different subtypes. In our study, an H4 AIV was isolated until 4.7 weeks, and an AAvV-6 and two AAvV-1 isolates were obtained, and therefore infectious, at the end of the analyses after 7.7, 4.6, and 10 weeks, respectively. These detections are consistent with other published results demonstrating that the persistence of avian avulavirus is consistently higher than that of the influenza viruses (4–6, 25).

It is very likely that other viruses that undergo digestive excretion were also present in the studied slurries but went undetected, as they are possibly difficult to isolate using the allantoic route in embryonated eggs. Further studies with different isolation methods would therefore be necessary to more fully assess the role of slurry as a vector contributing to the spread of duck viral diseases.

The physicochemical composition of slurries could significantly influence the persistence of infectious AIV particles (22, 28, 36, 48). The pH values measured prior to lime treatment in the four slurries used for spiking experiments varied between 6.1 and 7.3, which correspond to the optimal pH range to maintain AIV infectivity (18, 21, 22, 28–34). The chemical composition of the slurries studied here was determined (Table S1). However, differences in composition are difficult to interpret due to the limited reference values regarding duck slurry composition (53, 54). In fact, slurry is a mixture of feces, urine, litter, and feed residues, supplemented by washing water and rainwater (the latter is added when slurry tanks are not fully covered); their quantities and compositions are therefore highly dependent on farming practices. In future studies, it could be interesting to analyze the composition of slurries after lime treatment at pH 10 and pH 12, to determine the potential valorization of nitrogen before possibly using treated slurries as fertilizers on agricultural land.

In conclusion, this study was aimed at evaluating the efficiency of specific sanitization practices implemented in France. Whereas HP AIVs can be disseminated by different routes (respiratory aerosols and aerial transmission, as well as movement of contaminated equipment and materials), this study was designed to better assess the risk of AIV persistence associated with slurry, a common way to store duck feces under farming conditions. To minimize the risk of AIV dissemination through slurries from infected farms, international guidelines have been published (22, 55). In France, three methods are authorized and regulated, namely, (i) treatment in a methanization structure, (ii) treatment with lime so that a pH between 10 and 12 is maintained for 7 days, and (iii) natural sanitization of slurry by storage for at least 60 days before spreading in the fields (34). Maximum persistence of A/H5 HP AIV for 7 weeks in naturally contaminated slurry without treatment, as demonstrated in our study, provides experimental support for the 60-day interval, which is mandatory in France for sanitization by prolonged storage. Similarly, our experiments with spiked and lime-treated slurries showed that no infectious virus was recovered 1 week posttreatment, which also lends experimental support to the mandatory interval of 7 days after lime treatment defined in French regulations. However, the study also presents evidence that other viruses (e.g., AAvV-1) persisted beyond 60 days, suggesting that similar studies could be beneficial if other viruses need to be controlled in the field. Finally, the experimental protocol described here can be used to test the efficiency of new sanitization methods, such as chemical and biological products or physical treatments, and could also be adapted for use with other pathogens that undergo digestive excretion.

## MATERIALS AND METHODS

### Selection of duck farms.

**(i) AIV-negative farms.** Four AIV-free farms were selected based on their AIV-negative status, determined by testing duck flocks with nucleoprotein (NP)–enzyme-linked immunosorbent assay (NP-ELISA; influenza A virus antibody test; Idexx) on 20 blood samples and with M gene real-time RT-PCR (M-rRT-PCR) (described below) on 20 cloacal swabs and 20 tracheal swabs, according to the OIE manual (56). The farms were selected to represent the diversity of the different slurry types in duck production in France: one assisted-feeding farm with mule ducks, two with Pekin duck breeders, and one with Muscovy duck breeders (Table 2). All AIV-negative farms were located outside southwestern France and were sampled between March and May 2016.

**TABLE 2:** Characteristics of AIV-free farms and naturally infected farms

Farm type and slurry	Type of production	Department	Date of^*a*^:					Type of slurry tank
			Detection	Depopulation	Collection	D0^*b*^	Study^*c*^	
AIV free								
A	Breeding Muscovy duck	22, Côtes d’Armor						Concrete
B	Foie gras; assisted-feeding period	24, Dordogne						Concrete
C	Breeding Pekin duck	49, Maine-et-Loire						Concrete
D	Breeding Pekin duck	49, Maine-et-Loire						Concrete

Naturally infected mule duck								
1	Foie gras; before assisted-feeding period	40, Landes	29/12/2016	30/12/2016	05/01/2017	30/12/2016	1.7 wk after D0	Concrete, not closed
2	Foie gras; before assisted-feeding period	40, Landes	05/01/2017	07/01/2017	06/01/2017	06/01/2017	0.7 wk after D0	Geotextile membrane, closed
3	Foie gras; assisted-feeding period	32, Gers	12/12/2016	19/12/2016	12/01/2017	19/12/2016	4.1 wk after D0	Geotextile membrane, closed
4	Foie gras; assisted-feeding period	32, Gers	23/12/2016	29/12/2016	13/01/2017	29/12/2016	2.7 wk after D0	Geotextile membrane, closed
5	Foie gras; assisted-feeding period	65, Hautes Pyrénées	23/01/2017	26/01/2017	08/02/2017	26/01/2017	2.6 wk after D0	Geotextile membrane, not closed

a Given as day/month/year.

b For artificially contaminated and lime-treated samples, D0 was the day of artificial contamination and treatment. For naturally contaminated slurry samples, the first day of the kinetic study (D0) was estimated as the last day when fresh fecal material from infected ducks could have been delivered into the slurry tank at the farm level (D0 therefore corresponded to the date when slurry was collected if ducks were still present on the farm when slurry was collected or to the date when the ducks were depopulated if depopulation was carried out before slurry sampling).

c Beginning of the kinetic study in the laboratory.

**(ii) Naturally infected mule duck farms.** Five contaminated farms carrying out foie gras production, during or before the assisted-feeding period, were selected in collaboration with the district animal health authorities (Direction départementale de la protection des populations), based on their positive A/H5N8 HP AIV status, as determined according to the OIE manual (56). Characteristics of the sampled flocks are summarized in Table 2. All selected farms were located in southwestern France and were sampled between January and February 2017.

### Collection and storage of fecal slurry samples.

All samples were collected with the agreement of both farmers and their official referring veterinarians. All samples were collected before disinfection was implemented in the poultry houses connected to the sampled slurry tanks, in order to avoid possible recent release of disinfectant residues into the sampled slurry.

All samples were collected in accordance with biosafety recommendations for HP AIV outbreak situations. These measures included wearing a disposable protective suit, gloves, and a respiratory mask. Boots and vehicles were disinfected before and after each visit with an approved virucidal disinfectant (Virkon; Lanxess). All disposable equipment and materials used for sampling were sealed in a plastic bag and destroyed at the farming site by an approved company.

The slurry tanks where samples were collected had not previously been homogenized, in order to keep a possibly higher virus concentration close to the feces adduction duct. No previous sanitizing treatment had been carried out in the tanks in order to keep the titer of possibly infectious virus as high as possible.

For each slurry tank, two 1-liter samples were collected nearest to the adduction duct delivering the fresh droppings into the tank. The sampling location was selected so as to possibly sample the virus last delivered to the slurry tank (i.e., the virus most likely to still retain infectivity at the time of sampling). One liter was taken from the surface of the tank and one liter at a 2-m depth. For surface sampling, a 4-meter long telescopic pole was used, with a 1-liter plastic beaker attached to the end. A bag with a capacity of 1 liter, weighted and equipped with a nonreturn valve (HydraSleeve GSH 130; EON Products, Inc.), was used to collect deep samples.

The surface and deeper slurry samples were transferred to separate screw-cap sterile glass bottles with a capacity of 1.5 liters each. The bottles were sealed, and their outside surfaces were cleaned with soapy water and then disinfected via a spray of Virkon. The bottles were then packed in double plastic bags and transferred with paper towels to a water-tight cooler containing frozen ice packs. The samples were directly transferred to an approved AIV-diagnostic laboratory and immediately shipped to the BSL3 containment facility of the French National Reference Laboratory, without any intermediate freezing step.

One slurry sample per farm (AIV-free slurries or naturally contaminated slurries) was sent to the laboratory for storage at 5°C before processing (see below).

### AIV-free slurry samples.

**(i) Physicochemical characterization.** The characteristics and composition of slurry samples used for artificial contamination were determined using official methods from the International Organization for Standardization (ISO) or European norm (EN): pH (EN 12176), electrical conductivity at 20°C and 25°C (EN 27888), density and dry matter content (percent) (EN 13040), organic matter and ash content (percent) (EN 13039), organic carbon (percent) (ISO 10694), total nitrogen (percent) (Kjeldahl method) (EN 13654), ammoniacal nitrogen (percentage of total N) (alkalinization, distillation, and titration), organic nitrogen (percentage of total N), nitrate nitrogen (percentage of total N) (colorimetry), nitrite nitrogen (percentage of total N) (colorimetry), calcium (EN 11855), potassium (EN 11855), sodium (EN 11855), phosphorus (EN 11855), sulfur, and carbon/nitrogen ratio.

**(ii) Validation of AIV-free status.** The AIV-free status of each slurry used for the artificial contamination study was checked in two steps. First, cloacal swabs were collected from the ducks present in the farms and were tested with M-rRT-PCR. Second, the collected slurry samples were tested again with M-rRT-PCR and were then analyzed by inoculation into embryonated eggs to check for a lack of embryo toxicity (irrespective of infectious or chemical origin). Slurries selected for the study had negative results for all these tests (negative M-rRT-PCR in cloacal swabs and slurry and no embryo toxicity of slurry).

### Artificial spiking with A/H5N9 HP AIV and subsequent lime treatment.

All experiments involving infectious A/H5N9 HP AIV were performed under BSL3 containment conditions. Our BSL3 laboratory has been approved by the French National Agency for Medicines and Health Products Safety (ANSM) to use specific zoonotic avian influenza viruses (no. ADE-077572017-1).

**(i) Artificially spiked slurries.** AIV-free slurry samples were artificially contaminated with the A/H5N9 HP AIV A/duck/France/150236b/2015 produced as contaminated allantoic fluids derived from specific pathogen-free (SPF) hen eggs as described previously (8, 10). A previous study using slurries naturally infected with A/H5N9 HP AIV had revealed average *C_T_* values of 30.2 ± 3.7 in M-rRT-PCR (57). In order to obtain similar quantities of AIV genome in our experimentally spiked slurries on the first day of the experiment, preliminary tests were performed to determine which virus dose needed to be inoculated: each negative slurry was spiked with several doses of the A/H5N9 HP AIV and then tested with M-rRT-PCR. For each slurry, the dose corresponding to the required quantity of genome was selected. In practice, between 600 μl (for slurries A, C, and D) and 6 ml (for slurry B) of A/H5N9 HP AIV was inoculated into 600 ml of slurry, depending on the viral stock used. After spiking with the A/H5N9 HP AIV, the slurry-virus mix was vigorously stirred for a few minutes and was divided into three equal portions. The first portion was maintained without any treatment; the other two portions were processed immediately for lime treatment.

**(ii) Subsequent lime-treatment of artificially contaminated slurry samples.** The second and third portions of the spiked samples were treated by addition of 45% milk of lime [Ca(OH)_2_+H_2_O; Labat Assainissement], at room temperature for less than 10 min, under continuous stirring. pH was controlled with pH universal indicator strips (Fisherbrand pH indicator [7.0 to 14] paper sticks; Fisher Scientific), so as to achieve pH 10 and pH 12 in the second and third portions. Artificially contaminated slurry samples, either untreated or treated with lime, were stored at 5°C ± 3°C for the rest of the study. A positive control consisting of PBS artificially contaminated with A/H5N9 HP AIV, at the same dose as the tested slurries, was prepared and stored similarly. Negative controls consisting of noncontaminated PBS, noncontaminated slurry without lime treatment, and noncontaminated slurry with lime treatment at pH 10 and pH 12 were prepared. All negative and positive controls were stored as described above and were collected and tested according to the same protocol as the contaminated slurries.

### Kinetic study of A/H5 HP survival in duck slurry samples: process for experimentally spiked and naturally contaminated slurries.

For naturally contaminated slurry samples, the first day of the kinetic study (D0) was estimated as the last day when fresh fecal material from infected ducks could have been delivered into the slurry tank at the farm level. D0 therefore corresponded to the date when slurry was collected, or to the date when the ducks were depopulated (Table 2). For spiked and lime-treated samples, D0 was the day of artificial contamination and treatment.

All slurry samples, both naturally contaminated and artificially spiked, either lime treated or untreated, were subsampled weekly (5 ml), after rehomogenization by 5 min stirring and were then analyzed by virus isolation in embryonated eggs, as described below, until the virus had not been isolated for two consecutive weeks. All slurry subsamples and allantoic fluids extracted at the end of each isolation assay were also tested using M-rRT-PCR. For the study of naturally contaminated slurry only, and in order to confirm the identity of the possibly detected virus, H5-rRT-PCR was also performed from the same slurry subsamples and allantoic fluids samples.

### Detection of AIV genomes by rRT-PCR.

**(i) RNA extraction.** A 1-ml portion of each collected subsample and allantoic fluid was clarified by centrifugation (20,000 × *g*, 4°C, 10 min). Then, 200 μl of supernatant was collected, and viral RNA was extracted using the RNeasy minikit (Qiagen) following the manufacturer’s protocol. Briefly, 200 μl of supernatant was mixed with 600 μl of lysis buffer (RLT) containing 6 μl of β-mercaptoethanol (Sigma) and left to stand for 10 min at room temperature. After addition of 700 μl of stringent washing buffer (RW1; Qiagen) and 400 μl of 70% ethanol followed by mixing, the liquid was applied to a spin column in two steps. Finally, washing and elution (50-μl volume) were performed.

**(ii) M- and H5-rRT-PCR.** M-rRT-PCR was carried out using a commercial kit (TaqMan one-step rRT-PCR master mix; Applied Biosystems) and the Applied Biosystems (ABI) 7500 Fast real-time PCR system. M-rRT-PCR primers, probe, and cycle conditions were as described by Spackman et al. (57).

Because of the presence of other AIVs, samples from naturally contaminated slurries that were positive by M-rRT-PCR were subjected to H5-rRT-PCR. H5-rRT-PCR was carried out using the same kit and apparatus as for the M-rRT-PCR. H5-rRT-PCR primers, probe, and cycle conditions were as described by Slomka et al. (58).

Analytical protocols used for extraction and rRT-PCR were first validated using this unusual slurry matrix: equivalent results of detection with M-rRT-PCR and H5-rRT-PCR were obtained with both PBS and slurry contaminated with the same dose of H5 virus. All negative controls in PBS were confirmed as negative by M-rRT-PCR in all experiments based on naturally contaminated or experimentally spiked slurries. In addition, each sample tested was analyzed with M-rRT-PCR or H5-rRT-PCR using an internal positive control, to confirm the absence of amplification inhibitors. Each real-time RT-PCR run included a dilution series of an appropriate common RNA standard, controlling for the fidelity of the methods and allowing the comparison of results between different runs. The analytical sensitivity of the M gene rRT-PCR was previously estimated to be 200 and 700 RNA copies per reaction for the A/H5N8 and A/H5N9 viruses, respectively, corresponding to 1,000 and 100 50% egg infective doses (EID_50_) of the same viruses. The standard deviation of the M gene rRT-PCR was estimated to be a *C_T_* value of 1.2 (standard *C_T_* values between 31 and 33), and that of the H5 gene rRT-PCR was a *C_T_* value of 1.3 (standard *C_T_* values between 34 and 36) using a standard RNA in a series of 20 repeated assays.

### *In ovo* isolation and characterization of viruses.

**(i) *In ovo* viral isolation.** Virus isolation was performed according to the official method recommended for AIV (56). After centrifugation (1,000 × *g*, 10 to 15 min, 2 to 8°C) of 4 ml of each subsample, 1,200 μl of supernatant was treated for 15 min at 4°C with/ml of penicillin, 10 mg/ml of streptomycin, 0.25 g/ml of gentamicin, and 0.25 mg/ml of amphotericin B (Fungizone). Then, 200 μl was used to inoculated the allantoic cavity of 9- to 11-day-old SPF embryonated chicken eggs (ANSES, Ploufragan, France). Five eggs were inoculated for each studied sample. Eggs were incubated at 37°C ± 1°C with daily candling. After 5 to 6 days of incubation or when embryo mortality was observed, allantoic fluids were collected and their hemagglutinating activity (HA) was tested. The HA assays were carried out with 1% chicken red blood cells in PBS, according to the standard protocol (56). A second serial passage in eggs was carried out similarly by using pooled HA-negative allantoic fluids harvested at the end of the first passage. Virus isolation was deemed negative when no mortality or hemagglutinating activity in allantoic fluids was detected at the end of the second egg passage.

For each isolation assay, a negative-control test using only inoculation of PBS was simultaneously checked. All negative controls in PBS were confirmed as negative by egg isolation in all experiments based on naturally contaminated or experimentally spiked slurries. No embryo toxicity was observed after inoculation of uncontaminated slurry or milk of lime into control embryonated eggs.

**(ii) Confirmation of the identity of the isolated viruses.** Samples of fecal material from conventional poultry may contain a variety of infectious hemagglutinating viruses that could be reisolated in embryonated eggs; it was therefore important to confirm the identity of each virus isolated throughout the kinetic study.

*(a) Virological identification.* Hemagglutinating viruses were identified through standardized hemagglutination inhibition (HI) assays (56), using a panel of anti-avian avulavirus (AAvV) and anti-AIV monospecific sera. Antisera used in HI assays were prepared in the authors’ laboratory by immunizing SPF chickens with reference AAvV-1 to -9 except AAvV-5 and with inactivated H1 to H15 AIV antigens, either obtained from the AI European Union Reference Laboratory (APHA Weybridge, United Kingdom) or isolated in France (Table S4).

*(b) AIV molecular identification by sequencing.* The HA2 domain of the HA gene was amplified with a pan-HA RT-PCR (59) or sequences encompassing the cleavage site in the H5 gene were amplified by J3/B2a or Kha1/Kha3 H5-specific RT-PCRs (60).

PCR products were sequenced in both directions using the PCR primers and the dye terminator method (ABI Prism DyeTerminator cycle sequencing ready reaction kit; Applied Biosystems) on an automated DNA sequencer ABI 373XL (Applied Biosystems). Sequencing data were assembled and inspected with Mega software. Subtype determination was performed by BLAST analysis on the influenza virus resource database (61).

**(iii) Detection of non-AIV contaminating viruses.** *(a) Molecular identification of non-AIV contaminating viruses.* AAvV-1 occasionally detected during the course of the study was pathotyped by sequencing the region encoding the cleavage site of the F protein, using in-house RT-PCR with the following primers: NDV F (sense), TAGAAAAAACACGGGTAGAAGA, and NDV R (antisense), TTGGTWGCRGCAATRCTCTC. Sequencing was performed as described above. The sequence of the cleavage site is correlated with the virulence of AAvV -1 (62, 63).

*(b) Neutralization of viral contaminants.* During the study of naturally A/H5N8 HP AIV-contaminated slurry, some samples yielded viruses different from A/H5 HP AIV. Moreover, their *in ovo* propagation was still associated with significant detection of the H5 genome by H5-rRT-PCR. This situation suggested coculture of A/H5 HP AIV with another virus, the latter being more easily reisolated than A/H5 HP AIV. In order to check whether infectious A/H5 viruses were in fact present in such cases, a confirmatory isolation assay was implemented, preceded by viral neutralization of the non-A/H5 contaminating virus.

These viral neutralizations were performed by incubating an equivolume mixture of the studied allantoic fluid with a hyperimmune antiserum, monospecific for the virus to be neutralized (produced by ANSES, Ploufragan, France), for 75 min at 4°C. Sera used for neutralization belonged to the same panel of AAvV and AIV monospecific antisera as those used in HI (see above). After this neutralization step, virus isolation in embryonated eggs was performed as described above (56).

## Supplementary Material

Supplemental file 1

Supplemental file 2

Supplemental file 3

Supplemental file 4
